# Editorial: Interactions between pathogens and host immune system in patients with immunodeficiency: estimation from high-throughput sequencing

**DOI:** 10.3389/fcimb.2023.1200638

**Published:** 2023-05-16

**Authors:** Jinmin Ma, Gilberto Sabino-Santos

**Affiliations:** ^1^ BGI-Shenzhen, Shenzhen, China; ^2^ Department of Microbiology and Immunology, School of Medicine, Tulane University, New Orleans, LA, United States; ^3^ Center for Virology Research, Ribeirao Preto Medical School, University of Sao Paulo, Ribeirao Preto, Sao Paulo, Brazil

**Keywords:** immunodeficiency, high-throughput sequencing (deep sequencing), mNGS (metagenomic next-generation sequencing), pathogen diagnosis, COVID-19

In recent years, meta-genomics sequencing (mNGS) has enabled the unbiased simultaneous detection of thousands of pathogens without *a priori* knowledge of a hypothesis to diagnose potential pathogens. This technology has been widely applied for clinical purposes and is particularly effective in detecting pathogens in critical infectious cases, making it a powerful tool for complex contagious diseases. In the past, discovering new pathogens usually took months to years, such as HIV from 1979 to 1986 and the SARS virus in 2003 (Peiris et al., 2004). However, with mNGS technology, the discovery process can now be completed in just a few days, as demonstrated by SARS-CoV-2 in 2019 (Lu et al., 2020), which benefited from large-scale mNGS applications. Moreover, new disease-causing pathogens have been discovered, such as Pseudorabies Virus (Ai et al., 2018) and the arenaviruses (Li et al., 2015). These viruses were known to infect a broad range of species across the globe and were previously thought to be non-pathogenic to humans.

Immunodeficient populations are susceptible to opportunistic pathogens that can cause severe disease, even though they may be harmless to immunocompetent individuals. The application of mNGS for diagnosing pathogens in clinical samples has been ongoing for years, starting with the case report of infectious *Leptospira santarosai* in 2014 (Wilson et al., 2014). Patients with immunodeficiency pose significant challenges for infectious disease diagnosis since pathogen detection is a prerequisite for precision therapy. Therefore, this Research Topic focuses on (i) performing simultaneous pathogen detection and immune evaluation on clinical samples, (ii) exploring differences in response to different pathogens in distinct immunodeficiency types, and (iii) providing guidance for clinical targeting of drugs and precision therapy strategies. Immunodeficiency can arise from various factors such as genetics, transplantation, and HIV/AIDS. Infectious diseases require complex immune responses, which may lead to opportunistic infections with different symptoms in immunodeficient individuals. The 13 studies included in this Research Topic can be categorized into five groups: 1) Rare pathogen infection; 2) transplantation patients; 3) genetic susceptibility; 4) fungal infection, and 5) other opportunistic infections (as shown in **Figure 1A**).

**Figure 1:**
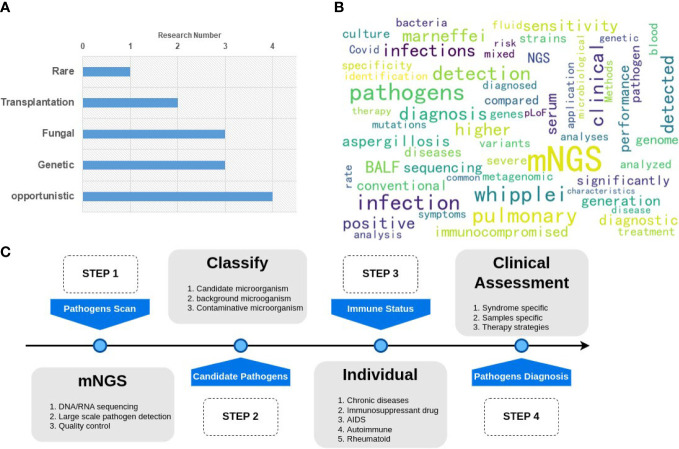
Graphical summary of the Research Topic. **(A)** the classification and number of the papers; **(B)** the hot keywords of the research. **(C)** A general pipeline of mNGS application for clinical pathogen detection.

In the category of rare pathogen infections, Zhou et al. reported the first instance of direct detection through mNGS of *Treponema pallidum* in patients with ocular involvement and typical MRI results. They suggest using mNGS as a potential supplementary tool for the diagnosis and differential diagnosis of neurosyphilis.

Transplant patients are at high risk of infection with long-term use of immunosuppressive drugs. This Research Topic includes studies on organ transplantation Liver transplantation (Huang et al.) and stem cell transplantation (Zhang et al.) patients. For the liver transplantation patient’s infection research, the author demonstrated that mNGS was superior (pathogen-positive rate: 64.3%) over traditional laboratory methods (pathogen-positive rate: 28.6%) for early identification and assisted in clinical decision-making for donor-derived infections. In addition, mNGS is a powerful technology to help the diagnosis of fever after stem cell transplantation, with the sensitivity of mNGS versus conventional microbiological at 91.2% vs. 41.2%, respectively, in peripheral blood samples.

Three studies observed individuals with autoimmune and genetically-related diseases to be susceptible to infections. One of the research papers identified the microbial agent responsible for causing pneumonia in patients with myasthenia gravis, an autoimmune neuromuscular disorder that affects neuromuscular junctions and leads to fluctuations in muscle weakness. Early infection discovery can lead to accurate antibiotic options and interventions when risk factors are present (Su et al.). Another study focused on the role of rheumatic diseases in complex infections, with the lungs being a common target for autoimmune-mediated injury. Metagenomic next-generation sequencing was a powerful complement to conventional methods in identifying pathogens in patients with rheumatic diseases (Jiang et al.). Finally, a study focused on the influence of rare genetic defects arising from inborn errors of immunity on COVID-19 severity. The study found that, in severe COVID-19-related patients, mutations were enriched in pathways related to tuberculosis, primary immunodeficiency, influenza, and HC-pLoF. This discovery identified candidate genes and pathways that can potentially be used as COVID-19 diagnostic markers to help distinguish patients at higher risk (Liu et al.).

Fungal infections are typically not found in individuals with standard immune systems but are more likely to occur in older individuals with compromised immune systems. One of the challenges in diagnosing fungal infections is the significant variation in fungi and their appearance in different samples. In this regard, mNGS-specific indicators can accurately and rapidly identify pathogens in patients with invasive fungal infections (IFIs) more effectively than conventional microbiological tests (CMTs), which has important clinical implications (Wang et al.). Other authors have shown that mNGS is a valuable and effective method for diagnosing pulmonary aspergillosis through bronchoalveolar lavage fluid (BALF) and blood samples (Bao et al.) but not for detecting Cryptococcus. For immunocompromised patients, these authors recommend using BALF detection when compared to tissue and cerebrospinal fluid (CSF) methods (Su et al.). Additionally, positive mNGS results for diagnosing cryptococcosis were found to be correlated with lower lymphocyte counts, higher IL-2, and higher serum antigen assays in immunocompromised patients.

Opportunistic pathogens can become pathogenic when the immune system weakens, or due to inborn errors of immunity, although they may be harmless to healthy individuals. For people living with HIV (PLHIV), *Talaromyces marneffei* can cause talaromycosis, and due to weak immunity, lead to significant morbidity and mortality if not diagnosed promptly. mNGS has been shown to be a powerful technique with high specificity and sensitivity for the rapid diagnosis of talaromycosis (Mao et al.). BALF samples analyzed by mNGS are a good option for early identification of *T*. *marneffei* in PLHIV with excellent performance in identifying mixed infections, enabling timely treatment, and potential mortality reduction. However, *T*. *marneffei* infection was first reported in patients with inborn errors of immunity with IL12RB1 gene mutation (Liu et al.), even in children who were not infected with HIV, which shows that susceptibility to *T*. *marneffei* appears to be related to genetics. *Tropheryma whipplei* is another opportunistic pathogenic bacterium associated with Whipple’s disease (WD). Using mNGS in a previous study (Lin et al.), the overall prevalence of *T*. *whipplei* was found to be 4.0 (70/1725). According to this largest *T*. *whipplei* cohort research, *T*. *whipplei* should be considered a potential contributing factor in some lung diseases, even in immunocompetent patients. Besides its high performance in pathogen identification, mNGS technology can also obtain genome information of the pathogen, enabling the analysis of mutations related to the severity of the disease. In this topic, two genomes of *Tropheryma whipplei* were assembled and characterized after mNGS pathogen identification (Lv et al.).

Regarding these 13 studies, all relevant keywords were analyzed in **Figure 1B**. It was found that the primary sample type used was BALF, and the diagnosis results in terms of sensitivity, specificity, and significance were the primary points of comparison between mNGS and culture or other traditional methods.

Although mNGS can detect a wide range of pathogens, clinical diagnosis requires additional individual information to make a final decision. Therefore, the entire pipeline for mNGS pathogen diagnosis should follow four steps, as shown in **Figure 1C**. Step 1 involves the pathogen scan, which includes sample sequencing and data analysis. Step 2 consists of the classification of microorganisms to identify candidate pathogens. Step 3 involves the estimation of individual immunity to assess the patient’s susceptibility to specific pathogens. Step 4 involves clinical assessment. A final diagnosis report is then generated based on the results of these four steps.

Despite this, generating a final diagnosis remains a challenging task due to the complexity of the interaction between pathogens and the human immune system. Therefore, further studies should address clinical issues such as pathogen colonization and infection. Additionally, in the future, using artificial intelligence methods to generate clinical pathogen reports automatically could promote more effectively the widespread application of mNGS.

## Author contributions

JM draft and final write the manuscript, GS-S reviewed and edited the manuscript. All authors contributed to the article and approved the submitted version.
